# Is deck C an advantageous deck in the Iowa Gambling Task?

**DOI:** 10.1186/1744-9081-3-37

**Published:** 2007-08-06

**Authors:** Yao-Chu Chiu, Ching-Hung Lin

**Affiliations:** 1 Department of Psychology, Soochow University, Taipei, Taiwan; 2 Institute of Neuroscience, School of Life Science, National Yang-Ming University, Taipei, Taiwan; 3 Laboratory of Integrated Brain Research, Department of Medical Research & Education, Taipei Veterans General Hospital, Taipei, Taiwan

## Abstract

**Background:**

Dunn *et al.* performed a critical review identifying some problems in the Somatic Marker Hypothesis (SMH). Most of the arguments presented by Dunn focused on the insufficiencies for replication of skin conductance responses and somatic brain loops, but the study did not carefully reassess the core-task of SMH. In a related study, Lin and Chiu et al. identified a serious problem, namely the "prominent deck B phenomenon" in the original IGT. Building on this observation, Lin and Chiu also posited that deck C rather than deck A was preferred by normal decision makers due to good gain-loss frequency rather than good final-outcome. To verify this hypothesis, a modified IGT was designed that possessed high contrast of gain-loss value in each trial, with the aim of achieving a balance between decks A and C in terms of gain-loss frequency. Based on the basic assumption of IGT, participants should prefer deck C to deck A based on consideration of final-outcome. In contrast, based on the prediction of gain-loss frequency, participants should have roughly equal preferences for decks A and C.

**Methods:**

This investigation recruited 48 college students (24 males and 24 females) as participants. Two-stage IGT with high-contrast gain-loss value was launched to examine the deck C argument. Each participant completed the modified IGT twice and immediately afterwards was administered a questionnaire to assess their consciousness and final preferences following the game.

**Results:**

The experimental results supported the predictions regarding gain-loss frequency participants choose the deck C with nearly identical frequency to deck A, despite deck C having a better final outcome than deck A. The "sunken deck C" phenomenon is clearly identified in this version of IGT which achieves a balance in gain-loss frequency. Moreover, the "sunken deck C" phenomenon not only appears during the first stage, but also during the second stage of IGT. In addition, questionnaires indicated that normal decision makers disliked deck C at the consciousness (explicit) levels.

**Conclusion:**

In the modified version of IGT, deck C was no longer preferred by normal decision makers, despite having a better long-term outcome than deck A. This study identified two problems in the original IGT. First, the gain-loss frequency between decks A and C is pseudo-balanced. Second, the covered phenomenon leads to most IGT related studies misinterpreting the effect of gain-loss frequency in situations involving long-term outcomes, and even leads to overstatement of the foresight of normal decision makers.

## Background

Damasio [1-4] extended the James-Lange theory with Somatic Marker Hypothesis (SMH) to not only explain the generation of subjective feeling but also the factors guiding decision making. Bechara et al. [5,6] then designed the Iowa Gambling Task (IGT) to demonstrate the operation of Somatic Markers in ventromedial prefrontal cortex (VMPFC) deficits and normal decision makers. In the serial studies conducted by Bechara et al. [5,7,8] normal controls gradually shifted their choice from the bad decks (A and B) to the good decks (C and D), but VMPFC deficits preferred the bad final-outcome decks (A and B) throughout the game.

In the original construction of IGT, bad decks A and B possessed relatively large gain-loss and disadvantageous final-outcome, while good decks C and D had relatively small gain-loss and advantageous final-outcome. Decks A and C contained five gains and five losses, while decks B and D contained nine gains and one loss in each circle of ten trials. The gain-loss frequency is balanced, with 14 gains and 6 losses between the bad (A and B) and good (C and D) decks in IGT (see Table 1).

**Table 1:** The gain-loss structure in the original IGT.

**IGT**	**A**	**B**	**C**	**D**
1	100	100	50	50
2	100	100	50	50
3	100, **-150**	100	50, **-50**	50
4	100	100	50	50
5	100, **-300**	100	50, **-50**	50
6	100	100	50	50
7	100, **-200**	100	50, **-50**	50
8	100	100	50	50
9	100, **-250**	100, **-1250**	50, **-50**	50
10	100, **-350**	100	50, **-50**	50, **-250**

11	100	100	50	50
12	100, **-350**	100	50, **-25**	50
13	100	100	50, **-75**	50
14	100, **-250**	100, **-1250**	50	50
15	100, **-200**	100	50	50
16	100	100	50	50
17	100, **-300**	100	50, **-25**	50
18	100, **-150**	100	50, **-75**	50
19	100	100	50	50
20	100	100	50, **-50**	50, **-250**

21	100	100,**-1250**	50	50
22	100,**-300**	100	50	50
23	100	100	50	50
24	100, **-350**	100	50, **-50**	50
25	100	100	50, **-25**	50
26	100, **-200**	100	50, **-50**	50
27	100, **-250**	100	50	50
28	100, **-150**	100	50	50
29	100	100	50, **-75**	50, **-250**
30	100	100	50, **-50**	50

31	100, **-350**	100	50	50
32	100, **-200**	100, **-1250**	50	50
33	100, **-250**	100	50	50
34	100	100	50, **-25**	50
35	100	100	50, **-25**	50, **-250**
36	100	100	50	50
37	100, **-150**	100	50, **-75**	50
38	100, **-300**	100	50	50
39	100	100	50, **-50**	50
40	100	100	50, **-75**	50

**Final Outcomes**	**-1000**	**-1000**	**+1000**	**+1000**

**Gain-loss Frequency (in average 10 tirals)**	**5 gains 5 losses**	**9 gains 1 loss**	**5 gains 5 losses**	**9 gains 1 loss**

SMH assumed that normal participants can achieve foresight by the help of somatic system; namely, they can obtain final benefit via implicit processing of emotion while performing the IGT. VMPFC deficits can not inhibit the preferences of such participants regarding the immediate large-gain decks A and B without the involvement of VMPFC function and the somatic system.

During recent years, the Iowa gambling task (IGT) has been become a critical task in research on affective function and decision-making under uncertainty. Bowman *et al*. [9] mentioned that the IGT has been applied in over 100 neurological and psychiatric studies. Recently, the IGT has also been utilized in neuroeconomics and psychology studies [10,11].

Nevertheless, Dunn* et al.*[12] conducted a global review for SMH and IGT and pointed out that IGT, skin conductance response (SCR) and somatic brain loops were three cornerstones in support of the Somatic Marker Hypothesis. Dunn *et al. *have summarized a significant body of inconsistent evidence demonstrating the inability of SCR and somatic brain loops to guide beneficial decisions. Notably, there have been few attempts to directly evaluate the core-task of SMH, namely IGT, with the exception of the "prominent deck B phenomenon" proposed by Lin and Chiu *et al.*[13].

Lin and Chiu *et al.*[13-15] pointed out a serious confounding in the IGT, and that Bechara et al. misinterpreted the result of IGT to fit the SMH. One critical piece of evidence is the "prominent deck B" phenomenon; specifically, even normal participants can not inhibit their preference for the high-frequency gain deck B with bad final-outcome. This phenomenon completely contradicts the principal assumption of the IGT. Lin and Chiu *et al.*[13-15] suggested that participants' behavioral decision was dominated by gain-loss frequency, not long-term outcome. In the study of Lin and Chiu *et al.*[13-15], participants preferred decks B, C, D to deck A. The high-frequency gain for decks B and D (nine gains and one loss) rather than deck A (five gains and five losses) is reasonable; however, the above prediction is against the basic assumption inherent in deck C (five gains and five losses) in IGT. Lin and Chiu *et al.*[13] explained the reason participants preferred deck C to deck A is due to that deck C contains fewer losses than deck A; for example deck C has five gains and five standoffs (rather than five losses) during the first ten trials. The five standoffs result from the setup of "paying $ 50 and earning $ 50" in the trial (five of ten trials). Participants therefore actually experienced 5 gains and 5 standoffs (standoff: net value is $ 0 within a trial) in deck C which possessed a superior gain-loss frequency to deck A (see Table 2). For an average of 40 trials of the IGT table, deck A contains 5 gains and 5 losses; however, deck C contains 6.25 gains, 2.5 draws and only 1.25 losses. Consequently, from this perspective, the gain and loss probability between bad deck A and good deck C is not equal as Bechara *et al*. suggested [5]. That is, the gain-loss structure between decks A and C is pseudo-balanced (e.g. based on the suggestion by Bechara *et al*., the gain-loss frequency of decks A and C was balanced with 5 gains and 5 losses; however, according to the net-value account, deck A still contains 5 gains and 5 losses, whereas deck C contains 6.25 gains, 2.5 draws and 1.25 losses over an average of 40 trials) in Iowa group's original proposal.

**Table 2:** The net value of each trial in the original IGT.

**IGT**	**A**	**B**	**C**	**D**
1	100	100	50	50
2	100	100	50	50
3	**-50**	100	**0**	50
4	100	100	50	50
5	**-200**	100	**0**	50
6	100	100	50	50
7	**-100**	100	**0**	50
8	100	100	50	50
9	**-150**	**-1150**	**0**	50
10	**-250**	100	**0**	**-200**

11	100	100	50	50
12	**-250**	100	25	50
13	100	100	**-25**	50
14	**-150**	**-1150**	50	50
15	**-100**	100	50	50
16	100	100	50	50
17	**-200**	100	25	50
18	**-50**	100	**-25**	50
19	100	100	50	50
20	100	100	**0**	**-200**

21	100	**-1150**	50	50
22	**-200**	100	50	50
23	100	100	50	50
24	**-250**	100	**0**	50
25	100	100	25	50
26	**-100**	100	**0**	50
27	**-150**	100	50	50
28	**-50**	100	50	50
29	100	100	**-25**	**-200**
30	100	100	**0**	50

31	**-250**	100	50	50
32	**-100**	**-1150**	50	50
33	**-150**	100	50	50
34	100	100	25	50
35	100	100	25	**-200**
36	100	100	50	50
37	**-50**	100	**-25**	50
38	**-200**	100	50	50
39	100	100	**0**	50
40	100	100	**-25**	50

**Final Outcomes**	**-1000**	**-1000**	**+1000**	**+1000**

**Gain-loss Frequency (in average 10 trials)**	**5 gains 5 losses**	**9 gains 1 loss**	**6.25 gains 2.5 standoffs 1.25 losses**	**9 gains 1 loss**

However, there is no direct evidence that the fewer loss (of deck C) are the main reason participants prefer deck C to A.

To obtain such evidence, this study provided a modified IGT (see Table 3). In this modified task, all gain-loss values were almost doubled in each trial to make the five losses of deck C visible, while keeping the final outcome the same as in the original IGT. This manipulation increased the contrast between gain and loss, and increased participant sensitivity to the embedded rule of each deck. As suggested by the Iowa group, the change in gain-loss frequency and immediate value have less effect on the participants' penetration to long-term benefit [5,16]. For example, Bechara *et al*. [16] modified the IGT by changing all "+" signs to "-" in each trial; thus, two good decks (+250) will become two bad decks (-250) and vice versa. In this version of the IGT, Bechara *et al*. found that final-outcome remains the primary guiding factor for decisions (namely, positive final-outcome decks were preferred by most normal subjects). Based on Bechara et al. serial finding, choice behavior is less influenced by other factors (e.g., gain-loss frequency).

If the proposal of Lin and Chiu *et al. *[13] is applied, the standoff is the critical factor for generating the differences between decks C and A, and participant preferences should be similar between decks A and C. Restated, the difference should be reduced because deck C had identical gain-loss frequency to deck A (5 gains and 5 losses) in this investigation. Nevertheless, if deck C is also selected more frequently than deck A in the modified IGT, the hypothesis regarding gain-loss frequency may require reconsideration.

**Table 3:** The gain-loss structure in the modified IGT.

**Deck Card Sequence**	**A**	**B**	**C**	**D**
1	200	200	100	100
2	200	200	100	100
3	**-50**	200	**-50**	100
4	200	200	100	100
5	**-350**	200	**-50**	100
6	200	200	100	100
7	**-150**	200	**-50**	100
8	200	200	100	100
9	**-250**	**-2050**	**-50**	100
10	**-450**	200	**-50**	**-650**

**Final Outcomes**	**-250 ($)**	**-250 ($)**	**+250 ($)**	**+250 ($)**

**Gain-loss Frequency**	**5 gains** **5 losses**	**9 gains** **1 loss**	**5 gains** **5 losses**	**9 gains** **1 loss**

## Methods

This investigation recruited 48 college students (mean age, 20.67 years old; SD, 1.23; age range, 19–22 years old) who were from different departments and participated in this experiment as a course requirement in psychology to perform this modified version of IGT. The position effect was counterbalanced by 24 card-position arrangements (e.g. ABCD, ACDB, ADBC ...DABC). In each case card-position was performed by two participants (one male and one female) to control gender differences. Furthermore, each participant was required to perform the same game twice to trace long-term participant preferences after the completion of first 100 trials in the modified IGT. This experiment adopted the original instructions for subjects performing the IGT [8,16]. All principal points of IGT instruction were adopted and are listed as follows:

" *1. In front of you on the screen, there are four decks of cards A, B, C, and D*.

*2. I want you to select one card at a time, by clicking on the card, from any deck you choose*.

*3. Each time you select a card from a deck, the color of the card turns red or black, and the computer will tell you that you won some money. I won't tell you how much money you will win. You will find out along the way. Every time you win, the green bar gets longer*.

*4. Every so often, however, when you click on a card, the computer tells you that you won some money, but then it says that you also lost some money. I won't tell you when you will lose or how much you will lose. You will find out along the way. Every time you lose, the green bar gets shorter*.

*5. You are absolutely free to switch from one deck to another any time you wish*.

*6. The goal of the game is to win as much money as possible and, if you find yourself unable to win, make sure you avoid losing money as much as possible*.

*7. I won't tell you for how long the game will continue. You must keep on playing until the computer stops*.

*8. You will get this $2000 credit (see the green bar) to start the game. At the end, we will see how much you won or lost. The red bar here is a reminder of how much money you borrowed to play the game*.

*9. It is important to know that the colors of the cards are irrelevant in this game. The computer does not make you lose money at random. However, there is no way for you to figure out when the computer will make you lose. All I can say is that you may find yourself losing money on all of the decks, but some decks will make you lose more than others. You can win if you stay away from the worst decks*." (Bechara *et al.*, 1999, p. 5474)

Following the two-stage game, each participant immediately completed a questionnaire regarding their final conscious preferences. The questionnaire asked subjects to recall the number of each deck they chose in the both games (total 200 trials), and, according to their present preferences, to allocate 200 trials for the four decks. While playing the game participants were informed that they were completely free and unrestricted to play as they wished, including the absence of any time restrictions. Additionally, participants were informed that the internal regulations (game structure and rules) were identical for both games.

## Results

### First Stage

**Figure 1:**
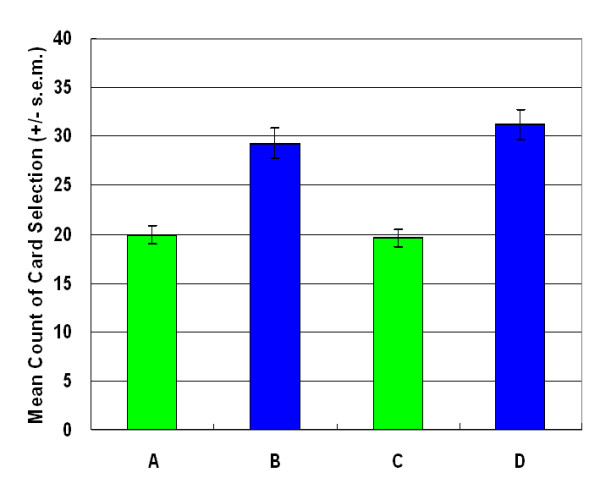
**Mean deck preference during first session**. The participants preferred decks B and D to decks A and C. The high-frequency gain decks (B, D) seem more attractive to normal decision makers. The final-outcome hypothesis of the Iowa group is invalidated by the "prominent deck B and sunken deck C" phenomenon.

The results of this investigation supported the hypothesis of Lin and Chiu *et al*. [13] and indicated that the participants preferred high-frequency gain decks B and D to high-frequency loss decks A and C during the first stage (Figure 1). A two-factor (final-outcome vs. gain-loss frequency) ANOVA (repeated measurement) was used for statistical testing of the four decks. Decks A and B (bad outcome]) were compared to decks C and D (good outcome), but no significant effects were observed in relation to the final-outcome (stage 1: *F *(1, 47) = .35, *p *= .55). In the gain-loss frequency domain, decks A and C (low-frequency gain) were compared to decks B and D (high-frequency gain), and the difference was significant (stage 1: *F (1, 47*) = 48.79, *p *< .01). Additionally, no significant interaction was identified between final-outcome and gain-loss frequency (stage 1: *F (1, 47*) = .59, *p *= .45).

**Figure 2:**
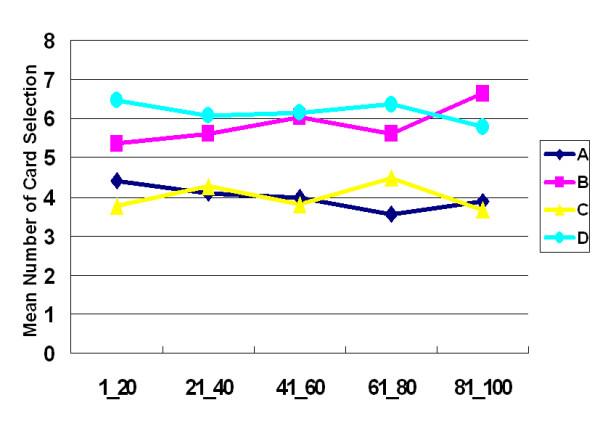
**Mean number of card selection in blocks during stage 1**. The learning curve between the advantageous decks (A, C) and disadvantageous decks (B, D) displays no cross over. Based on the suggestions of the Iowa group regarding final-outcome, participants should gradually shift their choice from decks A and B to decks C and D. However, this study does not support this proposal.

The learning curve also indicated that participants' choice behavior was occupied by the high-frequency gain decks (B and D) rather than the low-frequency gain decks (A and C) (Figure 2). Repeated-measurement ANOVA was performed to test the three factors, including final-outcome (good vs. bad), gain-loss frequency (high vs. low) and blocks (1 to 5) (see Table 4).

**Table 4:** Main effect and interaction of learning curve during stage 1

**Effect**	**F**	**Hypothesis df**	**Error df**	**P**
OUTCOME	0.35	1	47	0.56
FREQ	42.21	1	47	0.00
BLOCK	213.30	1	47	0.00
OUTCOME * FREQ	0.28	1	47	0.60
OUTCOME * BLOCK	1.13	4	44	0.35
FREQ * BLOCK	0.47	4	44	0.76
OUTCOME * FREQ * BLOCK	0.91	4	44	0.47

### Second Stage

**Figure 3:**
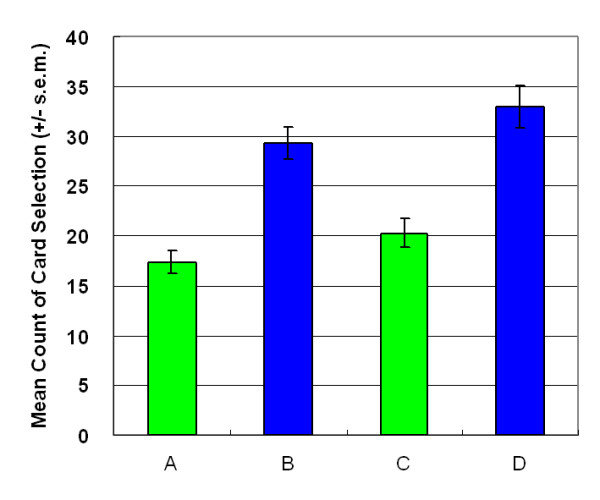
**Mean deck preference during second session**. This additional session aims to verify learning status after the game. The results replicate the finding of the first session, with participants being insensitive to the bad final-outcome of deck B and the good final-outcome of deck C. The present observation invalidates the suggestion of the Iowa group that normal decision makers can foresee final benefit.

Surprisingly, participant choice patterns were similar in both games. Participants were not inspired to select good final-outcome decks C and D after observing their performance (stage 2: *F *(1, 47) = 2.57, *p *= .12); however, high-frequency decks B and D were attractive to normal decision makers (stage 2: *F *(1, 47) = 41.50, *p *< .01) (Figure 3).

**Figure 4:**
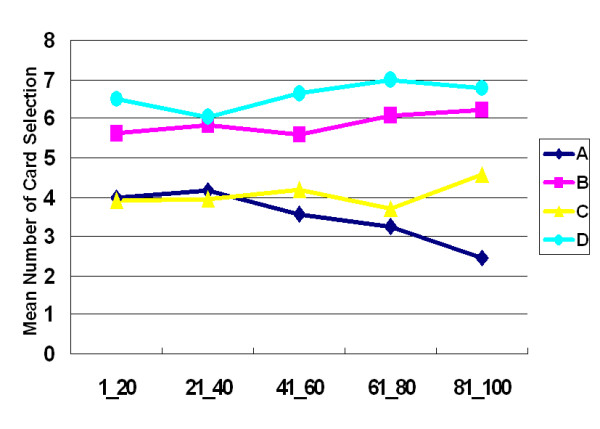
**Mean number of cards selected in blocks during stage 2**. The learning curve in stage 2 also replicated the findings from stage 1, with decks A and C exhibiting lower preference than decks B and D. Unexpectedly, participants preferred the high-frequency gain-decks (B, D) to the other two decks (A, C) even during the second encounter of IGT.

The interaction between final-outcome and gain-loss frequency was also not significant (stage 2: *F *(1, 47) = .04, *p *= .84). Moreover, the learning curve of stage 2 resembled that of stage 1, and no crossover was observed between decks A and C or decks B and D (Figure 4). Table 5 lists details of statistical testing between the three factors, final-outcome (good vs. bad), gain-loss frequency (high vs. low) and blocks (1 to 5).

**Table 5:** Main effect and interaction of learning curve during stage 2

**Effect**	**F**	**Hypothesis df**	**Error df**	**P**
OUTCOME	2.56	1	47	0.12
FREQ	41.55	1	47	0.00
BLOCK	0	1	47	1.00
OUTCOME * FREQ	0.04	1	47	0.84
OUTCOME * BLOCK	0.82	4	44	0.52
FREQ * BLOCK	0.80	4	44	0.53
OUTCOME * FREQ * BLOCK	0.77	4	44	0.55

### Questionnaire Stage

**Figure 5:**
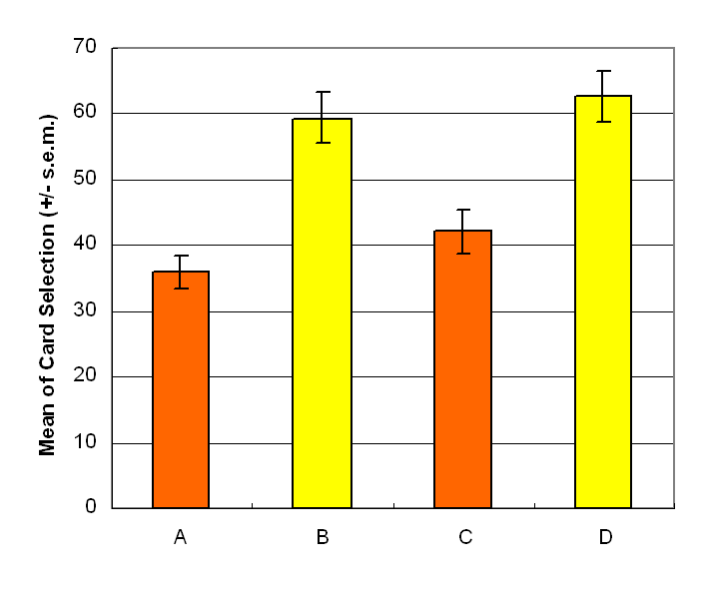
**Participant memory assessments in the modified IGT**. Following the two-session games, participants were asked to answer the following question: "*Please recall the number of trials you assigned for these decks during all 200 trials of the two-session game*". This result of questionnaire almost identified the behavioral choice pattern in figure 1 and 3. The questionnaire data confirmed that participant conscious memory is consistent with their choice pattern.

**Figure 6:**
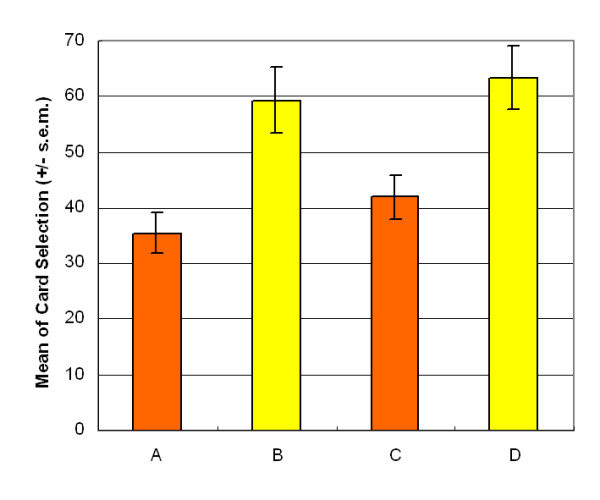
**Participant additional assignment of 200 trials after the two-stage game**. This question aims to verify participant final preferences regarding the four decks following the two-session game, and participants were asked to answer the following question: "*After playing the two-session game, please assign additional 200 trials to the four decks according to your preference*". Also, even when participants performed the IGT twice, they still preferred decks B and D to decks A and C.

After completing the two-stage game, subjects were required to recollect the number of trials they chose for each deck, thereby recording their preference on a conscious level. Furthermore, subjects were asked to distribute the 200 trials among the four decks to further identify their preference. The questionnaire data of memory assessment (Figure 5) and identification of further preference (Figure 6) indicated that following the two-stage IGT, participants are not only unable to foresee deck final outcome, but were also unable to enter the "hunch" state, even on level of consciousness.

## Discussion

The observations above supported the hypothesis of Lin and Chiu et al. [13] that participants preferred the deck C in the original IGT owing to gain-loss frequency, not final-outcome. Furthermore, the prominent deck B phenomenon can be identified again in this experiment (Figures. 1, 2, 3, 4). This study demonstrated that the original gain-loss structure of IGT is pseudo-balanced, particularly between decks A and C. Deck A possessed five gains and five losses in the domains of both loss account and net value (in each trial) (see Table 2), while deck C had five gains and five losses in the loss account domain, but contained five gains and five standoffs in terms of net value (in each trial). Actually, some studies identified the variant choosing-number of deck C, indicating (good final-outcome) that deck C is sometimes chosen less than (bad final-outcome) deck B [12,17-27]. Both "prominent deck B" and "sunken deck C" are inconsistent with the basic assumption of IGT and indicate that gain-loss frequency is the main factor guiding player (subjective feeling and decision-making) in these types of uncertainty game. Moreover, this study also observed that females are more sensitive to high-frequency gain than males; however, gender difference is not significant. The gender difference for frequency effect may be congruent with observation obtained by Overman *et al*. [21,28].

In fact, in the supplementary data of an IGT related study conducted by Maia and McClelland [29] who reported their observation on some subjects' account and indicated subjects' preference to decks B. Maia and McClelland's report listed as following, which is consistent with Lin and Chiu et al. [13] and present finding on "prominent deck B" phenomenon.

"*...Furthermore, given that on trial 40 she still seemed to think that deck B was a good deck ("because the winnings are still pretty high"), it also does not come as a surprise that she went back to deck B in the next period (only to lose $1,250 again). In fact, this pattern is not at all unusual. It often takes two $1,250 losses in deck B to convince participants to stay away from that deck. Even then, many (including participant 36) still go back to that deck a couple of times – sometimes because they understand that there is usually a series of "safe" $100 wins before the next $1,250 loss, and other times because they are higher risk-takers..*. " (Maia and McClelland, 2004, supporting text p. 11)

On the other hand, in Maia and McClelland's additional data also pointed out that (as listed below) actually, the immediate losses of deck C was too small and often ignored by subjects. Namely, the experimental control in terms of gain-loss frequency between decks A and C in original IGT were obviously pseudo-balanced. Participants preferred to choose deck C is not due to the good final-outcome, but due to the high-frequency gain and standoff (rather than deck A) in IGT.

"...*Between trials 40 and 70, she plays 26 out of 30 times from deck C, which is in line with her stated knowledge about that deck: "when I lose, I don't lose that much, and sometimes the wins and the losses cancel out" and "the losses aren't huge and I usually win or... you know..." (which we interpret as "I usually win or stay even"). (In fact, most participants find it easy to figure out that deck C provides them with a net gain..*." (Maia and McClelland, 2004, supporting text p. 11)

Notably the original presentation of IGT possesses a four-deck format [5] and in the Iowa group data deck B is less frequently selected. The chosen number of deck B is nearly equal to deck A. However, since 1994, the four-deck format of the IGT has mostly been substituted by the two deck format, simply involving a single good and a single bad category of decks. Unfortunately, most studies using the four-deck format were focused on the "prominent deck B" or "sunken deck C" phenomenon, and thus the Iowa group's "sunken" deck B (nearly equal to deck A) [5] appears unlikely to appear in any future studies, though notably one relatively recent study did contain such observations. The concern of the present study is identifying the confounding or methodological problem embedded in the original studies on the IGT. However, most IGT related studies utilized the summation of either good or bad decks to avoid the prominent deck B phenomenon and in confirming their findings regarding the original assumption of IGT. In studies where the "prominent deck B" applies, the issue is related to a pure academic argument, that is, an explanation is needed for why the "prominent deck B" phenomenon was not identified by over 100 research studies during the past 13 years. Serious questions need to be asked about why related research has always conformed to the hypothesis of the Iowa group. This issue rapidly requires careful reconsideration and exploration, before the IGT becomes a formal neurological or psychological assessment. The present study not only identified the misinterpretation of final-outcome in the original IGT, but also identified another powerful factor influencing decision makers' cognition in the face of uncertainty, namely gain-loss frequency [14,15]. Although, the "prominent deck B and sunken deck C" is incongruent with the original hypothesis of SMH, but both phenomena are more congruent with the behavioral decision literatures [30-34], affective neuroscience [35,36] and animal studies [37-40]. IGT is the core task in constructing the SMH; nevertheless, some critical variables of IGT suggested by Iowa groups are no more predictive. Additionally, not only have critical problems in IGT been identified, but physiological problems involving the somatic system of SMH have also been identified [20,29,41-48]. IGT has gradually become extremely important in affective neuroscience and neuroeconomics, and SMH has provided critical perspectives regarding the relationship between the emotional brain and decision-making. Nonetheless, the evidences presented here indicate that IGT and SMH should be carefully reevaluated before being accepted as convincing tool and theory.

In addition, there have increasing number of IGT related studies with normal subjects demonstrating that subjects preferred the high-frequent deck B rather than high-EV deck C [17,19,20,49]. Particularly, Wilder et al. [18] in 1998 have described the similar observation in control subjects. Their critical description for the IGT inconsistent result was listed as bellow.

*"... Moreover, both normal control subjects and schizophrenic patients appear to have been influenced by the immediate rewards, based upon both groups' choice of frequent rewards and infrequent penalties in Decks 'B' and 'D', rather than the long-term profits and losses associated with the task. The overall pattern of the two groups appeared to demonstrate that both were behaving according to the principle that large, infrequent penalties have less of an impact on long-term strategy than do frequent, small penalties. This being said, it should be noted that the groups still chose the 'good' decks (C and D) more than the 'bad' decks (A and B)...However, we did not find precisely the same pattern of deck preference with our normal control that Bechara et al. found... " *(Wilder *et al.*, 1998, p. 172)

Moreover, actually in MacPherson et al. [50] launched the Iowa gambling task to explore the function of ventromedial prefrontal cortex with comparing the age difference. However, this study obviously indicated that subjects preferred the high-frequent decks B and D rather than decks A and C as suggested as following.

*"...participants tend to show a preference for Decks C and D when performing the gambling task, whereas in this experiment participants showed a preference for Decks B and D. However, the current findings of a preference for Decks B and D have also been reported in healthy participants by Wilder, Weinberger, and Goldberg (1998). This pattern of performance indicates that participants were influenced by instant rewards rather than by the longer term profits and losses associated with the task. Participants seemed to believe that the small frequent penalties had a more negative impact on their long-term profit than the large infrequent penalties. Indeed, studies of risk-taking behavior and gambling have demonstrated that an individual's choices are not affected by the amount of money already won or lost on previous trials but the ratio of wins to losses (Greenberg & Weiner, 1966). Furthermore, when individuals are faced with ambiguous information and have to make decisions, they rely on the encoding of frequency information rather than the amount of reward (Hasher & Zacks, 1984)... " *(MacPherson *et al.*, 2002, p. 607)

In summary, the IGT is the core task of SMH for comparing decision behaviors between VMPFC patients and normal controls on behavior and psychophysiology. However, the "prominent deck B" and "sunken deck C" phenomena are not problems for the IGT that can be ignored. If the two phenomena are the true, the final-outcome will be no longer a predictive variable under uncertainty. Namely, decision-makers are unable to make foresighted decisions by cumulating and calculating the internal (somatic) markers, which represent external events precisely. Based on the inconsistent evidence [12,17-27] and present observations, we suggest that somatic markers may operate according to gain-loss frequency of external events, not final-outcome. This observation may be helpful in not only refining interpretations of IGT and SMH results, but also clarifying the relationship between choice behavior, reasoning and affective operation.

## Conclusion

This investigation demonstrated that the "prominent deck B" and "sunken deck C" phenomena are observable in this modified IGT. The hypothesis of Lin and Chiu *et al.*[13] regarding gain-loss frequency supported, but the basic assumption of the Iowa group regarding final-outcome was invalidated. The basic concept of the IGT is extremely original and the test is useful in simulating real-life decisions. However, the IGT contains some redundant procedures, confounding features, and problems in interpretation. These problems should be refined to make the IGT a truly useful assessment tool.

## Abbreviations

IGT: Iowa Gambling Task

SMH: Somatic Marker Hypothesis

VMPFC: Ventromedial Prefrontal Cortex

## Competing interests

The authors certify that the information presented in this study is complete to the best of our knowledge. The authors declare that they have no competing interests.

## Authors' contributions

YC and CH have made the equal contributions to thought, design, and data interpretation as well as drafting key concept of the manuscript and refining it critically.
